# The impact of R&D effort on business model innovation: Evaluating chain mediation through collaboration breadth and depth

**DOI:** 10.1371/journal.pone.0286715

**Published:** 2023-06-05

**Authors:** Shuting Chen, Dengke Yu

**Affiliations:** School of Public Policy and Administration, Nanchang University, Nanchang, China; Sichuan Agricultural University, CHINA

## Abstract

Drawing on a novel theoretical framework, we explored the impact of research and development (R&D) effort on business model innovation via external collaboration breadth and collaboration depth in sequence. We empirically analyzed a sample of 94 Chinese innovative enterprises by applying hierarchical regression analysis and chain mediation analysis. The results indicate that R&D effort positively influences business model innovation. The influencing mechanism is that R&D effort positively affects external collaboration breadth, which in turn positively stimulates external collaboration depth, and ultimately benefits the implementation of business model innovation. Therefore, the breadth and depth of external collaboration play a chain-mediating role. The study develops a new framework for understanding the relationship between R&D effort, external collaboration, and business model innovation. It combines enterprises’ internal behavior (R&D) and external behavior (collaboration) to establish an inside-out mechanism for predicting business model innovation. It enriches the theory of business model innovation. It also provides insights for managers and governments to optimize policies in innovation-driven development.

## 1 Introduction

With the advancement of the information economy and new technologies, such as big data, cloud technology, artificial intelligence and the internet of things [1], firms are facing unanticipated disruptive competition and new challenges. In today’s rapidly changing social and economic environment, firms are urged to change their processes, practices and operations, adopt particular business models or implement business model innovation to capitalize on emerging business opportunities and maintain development [2]. Recently, business model innovation has received an increasing amount of attention among practitioners and academics [3, 4]. In practice, many business model innovations are emerging and significantly changing the lifestyles of people and the business rules of most industries [5]. For example, Alibaba and Amazon, world-famous e-commerce giants, have achieved successes from their unique business model innovations, which break out the traditional offline shopping mode, enable people to shop without leaving home and influence others to follow. In academia, studies have shown that firms with faster growing operating margins often attach twice as much importance to business model innovation than their inefficient competitors [6]. Obviously, business model innovation, allowing firms to create novelty or improve efficiency that goes beyond product, process and technology innovation [7], is recognized as a source of sustainable competitive advantage and competitiveness [8].

Business model innovation is defined as “designed, nontrivial changes to the key elements of a firm’s business model and/or the architecture linking the elements” [9]. Although there is growing interest in business model innovation, previous studies have been relatively static and descriptive in nature. The focus of existing research mainly includes the drawing of a blueprint for the coherence between business model elements [10], the development of case studies involving business model innovation [11–13] and the exploration of the relationship between business model innovation and firm performance [14, 15]. Recently, more researchers have been devoted to exploring the antecedents of business model innovation. For instance, Yi, Chen and Li (2022) identified stakeholder ties and organizational learning as enablers for business model innovation [16]. Xu, He, Morrison, De Domenici and Wang (2022) found that entrepreneurial networks and effectuation are driving factors of business model innovation [17]. Despite these achievements, much more needs to be done to explore the field.

It is important to recognize the enabling factors through which firms can better generate new business models and maintain their competitiveness. Recent research has pointed out that R&D effort, as an internal factor, can create VRIN (valuable, rare, inimitable, and non-substitutable) knowledge and augment existing knowledge stock for firms, providing a crucial driving force of enterprise innovation [18]. However, thus far, prior studies have focused on exploring the impact of R&D effort on firm performance [19, 20] or technological innovation ability [21], but few have investigated its role in business model innovation. It is a gap. We therefore tried to fill this gap from an endogenous perspective. Considering the extroverted and open characteristics of business model innovation, we also deemed external links and open collaboration as important enablers, since external collaborations can help firms to break down traditional boundaries and allow useful external knowledge and information to enter into and flow across an organization [22–24]. In the field of external collaboration, its breadth and depth have been repeatedly employed to address research questions [25, 26]. Extant studies have shown that both are beneficial to enterprise innovation [19, 27]. Thus, we proposed that the two are also important antecedents of business model innovation.

According to the abovementioned analysis, R&D effort, collaboration breadth and depth may be relevant to business model innovation. However, they should not work independently. They are interrelated with each other in a convoluted manner. At present, the affecting mechanism between them is still unclear. Therefore, exploring the relationship between R&D effort, collaboration breadth, collaboration depth and business model innovation is necessary. We then tried to conduct an empirical analysis based on the sample data of Chinese top-ranking innovative enterprises to explore the inside-out mechanism of business model innovation driven by R&D effort. We drew on absorptive capacity (AC) theory to develop our framework [28, 29], in which collaboration breadth is regarded as potential absorptive capacity (PAC) and collaboration depth is regarded as realized absorptive capacity (RAC). On the basis of a literature review, our assumed logic can be described as follows. The investment and activities of R&D could promote PAC, and then the accumulation and transfer of PAC would strengthen RAC, which ultimately plays an important role in the change of an enterprise’s business model. Following such logic, we constructed an R&D-PAC-RAC-Innovation framework and built a chain mediation model to test the impact of R&D effort on business model innovation. We therefore addressed the following research questions:

RQ1. Does R&D effort positively affect business model innovation?
RQ2. Does collaboration breadth positively affect collaboration depth?
RQ3. How does R&D effort indirectly affect business model innovation via the mediators of collaboration breadth and depth?

In doing so, our present study makes three important theoretical contributions to the literature. First, this study explores the relationship between R&D effort and business model innovation, which extends the research on the antecedents of business model innovation and enriches the research on the outcomes of R&D effort. Second, it opens the black box of the relationship between collaboration breadth and depth, thereby contributing to the literature on external collaboration. Finally, based on the R&D-PAC-RAC-Innovation framework, it proposes two chain mediators of collaboration breadth and collaboration depth to clarify the internal influencing mechanism of R&D effort on business model innovation to strengthen the understanding of business model innovation under the background of firms’ R&D effort.

The remainder of this paper is structured as follows. Section 2 presents our theories and hypotheses. Section 3 describes the methodology, including the introduction of sample, data collection, measures and statistical techniques, following which Section 4 outlines the results of empirical analysis. Finally, Section 5 discusses theoretical implications, practical implications, limitations and future research and presents a short conclusion.

## 2 Theories and hypotheses

### 2.1 Theories

Currently, the business environment, which is characterized by fierce competition, fast technological change and strong market turbulence, compels most firms to innovate their business models to improve their adaptive capability [30]. Business model innovation is defined as a new-to-the-firm change in at least one out of three business model dimensions: (a) a firm’s value creation, (b) a firm’s value proposition, and (c) a firm’s value capture [31]. Value creation addresses how and by what means firms create new value along the value chain using their resources and capabilities embedded in intra- and inter-organizational processes [32]. Value proposition defines the range, nature and features of the offered products and services and the conditions at which these are provided [33]. Value capture answers the question of how value proposition is converted into profits in a sustainable way [34].

For firms operating in extremely turbulent environments, their sustainability relies heavily on the core competence and dynamic capability driven by continuous innovation. As the foundation of different kinds of innovations, R&D effort, including investments in R&D human resources, R&D spending and R&D equipment, plays an important role in the sustainable growth of firms [35]. The implementation of business model innovation should be carried out on the basis of R&D effort without exception since the creative thinking and absorptive capability that are essential to business model innovation need to be gradually built and improved through the training process of continuous R&D activities.

According to open innovation theory, collaboration with external organizations is crucial to a firm’s business model innovation, which is characterized by open principles and resource integration [23, 36, 37]. External collaboration is access that enables firms to acquire non-redundant knowledge and capabilities residing outside their organizational and technological boundaries [38, 39]. In prior literature, the construct was classified by two dimensions, i.e., breadth and depth [25]. Collaboration breadth is defined as the number of external sources that firms rely upon in their innovative activities, ranging from narrow to broad collaboration as external partners increase. In contrast, collaboration depth is recognized as the extent to which firms draw deeply from different external sources, ranging from surface to deep collaboration as collaborative interactions intensify [19, 25, 40]. The two reflect different kinds of capabilities of firms in the function of external knowledge absorption and resource integration.

### 2.2 R&D effort and business model innovation

The tacit nature of technological knowledge and the risks related to the loss of technological competitiveness require internal efforts in R&D activities [19]. With the advance of the knowledge-based economy, R&D effort becomes more essential for enterprises to carry out business model innovation, which is reflected in three aspects. First, the high attention on R&D promotes firms to develop new technologies and products, in turn stimulating business model innovation. However, the economic value of a technology or product remains latent until it is commercialized in some way via a business model [37]. Firms should change their existing business models to better capture the value within the new technologies or products. Next, R&D effort improves the development of technical human capital and is thus beneficial to changing the existing business model. Resource-based theory emphasizes the important role of R&D personnel knowledge and capabilities in the development of firms’ core competitiveness and sustainable competitive advantage [41, 42]. R&D human capital supports and participates in the implementation of business model innovation. Additionally, R&D effort increases opportunities for firms to embrace new things, providing a solid foundation for business model innovation [24]. To achieve R&D goals, firms need to keep track of the changes in customer demand, obtain cutting-edge knowledge, and communicate and cooperate with external organizations. These actions help firms to catch on the status quo and keep up with the trend of the external environment to find the optimal direction for business model innovation.

Several studies have provided empirical evidence for the positive impact of R&D effort on business model innovation. For example, Zouaghi et al. (2018) suggested that R&D intensity and R&D human capital are recognized as vital driving factors for innovations new to the market place and the firm [19]. Leung and Sharma (2021) revealed that R&D effort plays an important role in a firm’s sales, profitability and value. [20]. Therefore, we proposed the following hypothesis:

H1: R&D effort has a positive impact on business model innovation.

### 2.3 R&D effort and collaboration breadth

We argued that R&D effort stimulates the expansion of a firm’s external collaboration breadth due to three effects. First, the high attention on R&D leads a firm to devote itself to knowledge learning and collaborative innovation; thus, a firm’s effort on R&D would increase the invested financial resources that are essential to external collaboration. According to the corporate financial system, a part of R&D expenditure can be used for the construction of external collaboration networks and strategic alliances, as well as specific activities such as communication, learning and training, which greatly raise the collaboration breadth of firms. Collaborative R&D networks need a lot of money to support them [43, 44]. Park, Chen and Gallagher (2002) empirically demonstrated that firms with rich financial resources are more likely to increase external collaborations [45]. In practice, we also observed that large and medium enterprises with abundant R&D expenditure tend to carry out more collaborative innovation activities with universities, research institutes and leading customers. Second, firms making more R&D efforts explore more new technological and market opportunities, which stimulates them to network more collaborators to make joint investments and develop potential value. Because R&D effort generally focuses on projects far from the market and with technological challenges rather than typical execution [46], the resources needed to take the discovered opportunities may not reside within firms, but are scattered across diverse organizations. Hence, firms that successfully exploit the opportunities need to cooperate with various partners to access and integrate heterogeneous resources [47], which are supported by and back stimulate the construction of external collaboration networks. Finally, R&D effort, through enlarging the knowledge base, helps firms expand their external collaborations. On the basis of R&D effort, firms can obtain new experience, knowledge and information [21], which enhance their capabilities to identify, assimilate and apply external knowledge and then improve the possibility of cooperation with a variety of external organizations. In other words, R&D effort enriches a firm’s knowledge stock and diversity, and the latter increases the competence and attractiveness of the firm in the development of collaborations [28, 48].

Some studies can support our argument. Cerulli, Gabriele and Potì (2016), for example, showed that a company’s R&D input can increase the likelihood of external collaboration with various types of partners [49]. Chapman, Lucena and Afcha (2018) provided strong evidence for the significant positive impact of R&D subsidies on firm external collaboration breadth based on data analysis of Spanish firms [48]. Therefore, we advanced the following hypothesis:

H2: R&D effort has a positive impact on collaboration breadth.

### 2.4 Collaboration breadth and depth

By reviewing the extant literature, it can be clearly seen that collaboration breadth and collaboration depth are generally regarded as two parallel concepts [22] and little research has explored the internal relationship between them. In view of the research omission, further examination of this issue is required.

Based on this attention-based view, a firm can be considered as a system that structurally distributes attention [50–52], where attention refers to the capability to process different information sources and simultaneously extract the information that is useful for certain tasks [53]. The view acknowledges that managerial attention is a firm’s most valuable resource. Decision-makers in firms are therefore supposed to “concentrate their energy, effort and mindfulness deeply on a limited number of issues and tasks” [50]. Following this view, we proposed that as external collaboration breadth increases, decision-makers in firms are exposed to increasingly more external information so that they clearly know their current situation covering what firms currently lack and need in the future. This knowledge can enable them to focus on the selection and development of the most valuable collaborations according to firms’ status quo [50]. In addition, with the expansion of collaboration breadth, the marginal value of superficial collaboration would be diminishing and decision-makers may find it more difficult to deal with the booming knowledge [26]. The pressure of information overload attributed to broad collaboration would compel decision-makers to narrow their attention to strategic alliances with VRIN characteristics. Based on the above analysis, we proposed that as the breadth of collaboration increases, the depth would also subsequently increase. Therefore, we proposed the following hypothesis:

H3: Collaboration breadth has a positive impact on collaboration depth.

### 2.5 Collaboration depth and business model innovation

Some prior research has confirmed that external collaboration even plays an irreplaceable role in facilitating a firm’s internal innovation process [25, 54]. In this study we wanted to further prove that deep collaboration with external organizations can effectively boost business model innovation. First, deep collaboration provides enterprises with access to external heterogeneous information and knowledge [22], which lays a foundation for the integration of resources and capabilities required by the process of business model innovation. Knowledge absorption and integration help enterprises generate new thoughts and ways to develop new business models with novelty and efficiency [55]. Second, deep collaboration experience cultivates trust between the enterprise and its partners [22]. Trust serves would serve as a crucial coordination mechanism that improves the accurate understanding of newly acquired external information, guarantees technological transfer without resistance, and achieves timely knowledge sharing and substantive cooperation [56], thereby contributing to the effective implementation of business model innovation.

Collaboration depth is generally divided into three dimensions, i.e., vertical, horizontal and competitor collaboration [41, 57], which have different benefits for business model innovation. The strategic alliance with suppliers and customers enables enterprises to gain updated information from market and industry, pool complementary resources and improve learning routines [58]. Clearly, these actions can assist enterprises in developing efficiency-oriented business models [59]. Moreover, the close connection with lead users would make enterprises rapidly know the changing demand for new products with novel functions, further helping enterprises to improve the novelty of their business models [41]. In many knowledge-extensive industries, collaboration with competitors is generalized since any one enterprise cannot undertake the huge cost of technological innovation and the development of a novel business model. Deep collaboration with competitors plays the roles of risk sharing, information and resource sharing, and joint monopolization in business model innovation [24]. Finally, deep collaborations with governments, universities, consultants and others enable firms to efficiently obtain comprehensive and heterogeneous knowledge required by market development [19]. Such collaborations could also enhance the public’s identity with firms and improve their brand images, which promote the success of new business models. Following the above analysis, we proposed the third hypothesis:

H4: Collaboration depth has a positive impact on business model innovation.

### 2.6 The chain-mediating effect

Martínez-Sánchez, Vicente-Oliva and Pérez-Pérez (2020) proposed a new theoretical framework along with the R&D-AC-Innovation link [60]. According to their statement, absorptive capacity (AC) was the key mediator between R&D and innovation. In other studies, as an essential ability to identify, assimilate, transform, and exploit external knowledge, AC was recognized as an important component of corporate competence [61, 62]. When firms make efforts in R&D, the absorption of external knowledge can facilitate innovative activities and enhance innovation capability and efficiency. In a previous body of knowledge, AC was often divided into PAC and RAC [29]. Among them, PAC refers to the capability to acquire and assimilate external knowledge, whereas RAC refers to the capability to transform and exploit external knowledge [29]. They are indispensable in a firm’s process of exogenous growth [63]. However, there is a progressive relationship between the two in logic, i.e., PAC forms the premise of RAC [64]. We therefore expanded the R&D-AC-Innovation link and tried to develop an R&D-PAC-RAC-Innovation framework for our study.

In prior literature, external collaboration was summarized as a type of absorptive capacity. Broad collaboration means the high possibility of enterprises to access external knowledge but not always the effective exploitation of knowledge. Hence, it is exactly a kind of PAC. In contrast, collaboration depth reflects the degree of an enterprise’s effective utilization of external knowledge. It is therefore a kind of RAC. As the framework of R&D-PAC-RAC-Innovation revealed, firms making efforts on R&D would obtain financial support, discover new opportunities and lay a solid foundation of resources and capability to collaborate with various external organizations [48] and then gradually focus their limited attention on part of external organizations that can bring them benefits and opportunities to better realize their targets involving performance, innovativeness and sustainability [26, 50]. That is, collaboration breadth triggers collaboration depth, and the latter could enhance the firm’s capability of business model innovation by building trust, obtaining knowledge and developing value nets [1, 22]. In summary, we naturally proposed the following hypothesis:

H5: Collaboration breadth and depth play a chain-mediating role in the relationship between R&D effort and business model innovation.

**Fig 1:**
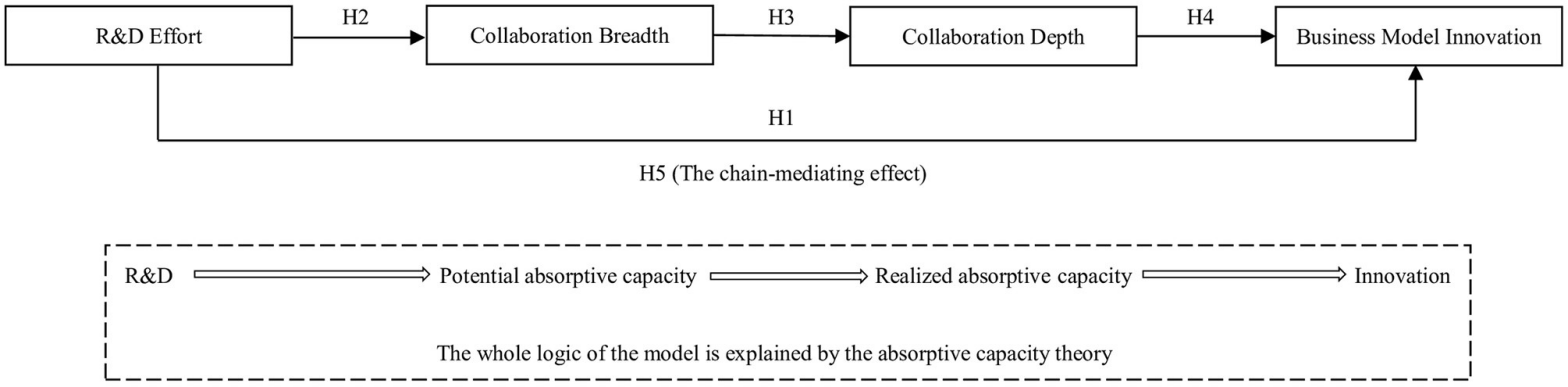
The conceptual research model of the study.

Fig 1 shows the conceptual research model of the study.

## 3 Methodology

### 3.1 Sample

Strategy&, part of the PwC network, released the 2018 Global Innovation 1000 study, which analyses the world’s top 1000 listed corporations with the highest R&D expenditures, and which account for 40% of the total global R&D expenditure. The Global Innovation 1000 study shows that R&D expenditures increase in every region of the world, but most notably in China, where they rise 34.4 percent over the previous year.

We collected the data of Chinese enterprises ranked in Global Innovation 1000 for three reasons. First, these enterprises are in line with the innovation-driven development strategy advocated by the Chinese national government. To some extent, this means that they are of great significance to the development of emerging countries. Second, most scholars focus on the technological innovation of such innovative enterprises but always neglect their excellent performance in business model innovation. Third, compared with small and medium enterprises, they generally have sufficient resources and strong capabilities to support their frequent collaborations. Moreover, their disclosed information is open and transparent, making it convenient for data collection.

To ensure the completeness and reliability of our data analysis, the following criteria were followed in the process of sample selection. First, we selected 175 Chinese companies in the 2018 Global Innovation 1000 study. Second, considering the consistency of the data structure, we excluded 76 companies that were listed in Hong Kong, Taiwan and the United States. Third, we excluded 5 companies with missing information. Finally, 94 Chinese companies listed on the Shenzhen and Shanghai Stock Exchanges (A share) were selected.

Table 1 displays the characteristics of our sample. The industries represented are information technology (26.6%), industrials (28.7%), materials (17.0%), consumer discretionary (21.3%), healthcare (4.3%) and energy (2.1%). The companies comprise 60.6% state-owned firms and 39.4% non-state-owned firms. Among them, 17.0% engaged less than 10000 employees, 42.6% between 10001 and 30000, 19.1% between 30001 and 50000, and 21.3% more than 50000. In addition, the sample aged 6–10 years accounts for 7.4%, 11–15 years for 12.8%, 16–20 years for 40.4%, 21–25 years for 31.9% and more than 25 years for 7.4%.

**Table 1:** Samples characteristics.

Variables	Category	Number (N)	Percentage (%)
Age	6–10 years	7	7.4
	11–15 years	12	12.8
	16–20 years	38	40.4
	21–25 years	30	31.9
	>25 years	7	7.4
Firm size	1–10000	16	17.0
	10001–30000	40	42.6
	30001–50000	18	19.1
	>50000	20	21.3
Ownership	State-owned	57	60.6
	Non-state-owned	37	39.4
Industry	Information Technology	25	26.6
	Industrials	27	28.7
	Materials	16	17.0
	Consumer Discretionary	20	21.3
	Healthcare	4	4.3
	Energy	2	2.1

### 3.2 Data collection

The data collection proceeded in two stages. In the first stage, we built composite scales for external collaboration and novelty-centered business model innovation, and we identified and measured the relevant items on the basis of a content analysis of company information (for details, please see **Appendices 1–3 in** S1 File). In the second stage, the data of the rest of the variables, including R&D effort, efficiency-centered business model innovation and control variables, were collected from the China Stock Market & Accounting Research Database (CSMAR Database).

In recent years, the panel method has been increasingly used [65, 66]. In this study, we set up a panel composed of 3 members, including 1 professor and 2 doctoral students. First, the professor carefully selected the panelists from his total team members by requiring them to submit an abbreviated test survey on a randomly chosen sample company to display their understanding of external collaboration and novelty-centered business model innovation. After the selection, 2 doctoral members jointly read the information, announcements and documents of the sample companies, became familiar with the details of the external collaboration and novelty-centered business model innovation of all sample companies, and then developed the measurement scale based on the consensus. Next, the professor trained them as expert raters in data collection and analysis. The raters were provided with written guidelines on the proper way to address survey items. Data sources included annual reports, social responsibility reports, investment analysts’ reports, company news, websites and other announcements of those companies from 2016 to 2020. The process took every rater approximately six months from October 2020 to April 2021. The lack of readily available data about external collaboration and novelty-centered business model innovation obliged us to collect primary data and construct a unique dataset. Finally, we evaluated the consistency by conducting a pairwise comparison of the two raters’ scores, yielding a Pearson correlation coefficient of 0.929 (p<0.01). For the items with discrepant scores, two raters discussed with each other and reached a consensus under the guidance of the professor. All initial differences were resolved, so the final consistency was 100%.

The data of other variables were drawn from the CSMAR Database, which is a research-oriented database in the economic and financial field compiled by Shenzhen CSMAR Data Technology Co., Ltd. The database reflects the financial conditions of China and follows the professional standards of authoritative databases such as CRSP, COMPUSTAT, TAQ and THOMSON. The data of these quantitative variables came from 2018. To control the influence of extreme values, the data collected from CSMAR have been winsorized.

### 3.3 Measures

#### 3.3.1 R&D effort

R&D effort has been extensively used in innovation research as an input variance [5, 19]. In this study, we measured it by a firm’s R&D expenditure as a proportion of the firm’s operating income [60].

#### 3.3.2 Collaboration breadth

Following Laursen and Salter (2006, 2014) [25, 67] and Dong and Netten (2017) [26], we constructed collaboration breadth as the combination of nine sources of external knowledge for business model innovation: 1) supplier, 2) customer, 3) competitor, 4) government, 5) university and research institution, 6) consultancy firm, 7) venture capital investment firm, 8) trade fair and exhibition, and 9) others. For each source, we used a three-point scale to indicate the scope of collaboration (1 = no or low degree, 2 = medium degree, 3 = high degree) (see **Appendix 1 in** S1 File for details). On the basis of the initial score, we further coded each source as a binary variable, where 1 represents that the collaboration width of a source is medium or high (2 and 3) and 0 represents that the source is not used or its collaboration width is low (1). Finally, the variable values of the nine sources were added up to measure the total level of collaboration breadth. Obviously, the value interval of the construct is [0, 9]. It was valued as 0 when all knowledge sources were not used or had low collaboration breadth, and valued as 9 when all sources had medium or high collaboration breadth.

#### 3.3.3 Collaboration depth

Following Laursen and Salter (2006) [25] and Dong and Netten (2017) [26], we defined collaboration depth as the intensity of collaboration with each source of external knowledge. A three-point scale was used to indicate the intensity of collaboration (1 = no or low degree, 2 = medium degree, 3 = high degree) (see **Appendix 2 in** S1 File for details). Similar to collaboration breadth, each source was further coded as a binary variable, where 1 represents that the certain external knowledge source is used to a high degree (3) and 0 reflects that it is not used, or only to a low or medium degree (1 and 2). The nine dummies were added up so that each of our sample companies could obtain the score of the depth variable, ranging from 0 to 9, where 0 indicates no intense use of any external knowledge source, and 9 indicates intense usage of all 9 sources.

#### 3.3.4 Business model innovation

We measured business model innovation from two dimensions, i.e., novelty-centered and efficiency-centered ones. Their scores were evaluated by the following different methods. Business model innovation was comprehensively measured by the mean value of their scores after data preprocessing for novelty-centered and efficiency-centered ones into the [0,1] interval.

*3*.*3*.*4*.*1 Novelty-centered business model innovation*. We independently developed a new scale of novelty-centered business model innovation. Four items, reflecting the new R&D system, new manufacturing platform, new sales model and new customer service system, were used to measure it. Considering the difficulty of the detailed measurement in an objective way, we deemed the use of perceptual measures obtained by our raters [65, 66]. The items were quantified on a five-point scale (see **Appendix 3 in** S1 File for details). After coding, their scores were aggregated and averaged for the final score.

*3*.*3*.*4*.*2 Efficiency-centered business model innovation*. We measured efficiency-centered business model innovation from the dimensions of value creation, value proposition and value capture in our study [31] (see **Appendix 4 in** S1 File for details). First, capital utilization ability and debt paying ability are the key elements in the process of value creation, so we selected the current ratio, equity-to-debt ratio and debt coverage ratio to measure value creation. Second, operating capacity is an essential factor in the value proposition process, which involves the turnover of goods, capital and assets. Thus, we used inventory turnover, accounts receivable turnover and total assets turnover to evaluate the value proposition. Third, it is very important for a firm to have profitability and growth ability in the process of value capture. Therefore, its measurement was composed of three financial indicators, i.e., net profit growth rate, operating income growth rate and operating profit ratio.

The entropy method is a widely used objective weighting method. It determines the weight of indicators by the variational degree of the dataset [68]. The TOPSIS method, based on the rule that the chosen alternative should have the longest distance from the negative ideal solution and the shortest distance from the positive ideal solution, is also a widely used evaluation method [69]. The negative ideal solution always maximizes the cost criteria and minimizes the benefit criteria, while the positive ideal solution is the opposite [70]. In this study, we combined the two methods and built an entropy-based TOPSIS model [71] to comprehensively assess efficiency-centered business model innovation.

#### 3.3.5 Control variables

To account for the effects of extraneous variables, we included firm ownership, firm age, firm size, firm location, industry, financial leverage and operating leverage as alternative explanations for business model innovation.

In China, firm ownership is an important factor that influences a firm’s strategy, operation and performance [22]. We measured it by a dummy variable that controls for potential variations between state-owned enterprises (coded as 1) and private-owned, foreign-owned or other types of enterprises (coded as 0).

Firm age also plays a role in a firm’s propensity to adopt business model innovation as it affects the flexibility of strategy and the willingness of knowledge absorption [72]. It was measured by the number of years since the firm was officially established.

Some scholars have argued that firm size matters for innovation, because large firms tend to have more resources required by innovative projects [73]. However, other scholars insisted that larger firms are more prone to organizational inertia, thereby hindering change processes [74]. To control the possible effect, firm size measured by the natural logarithm of employee scale was set as a control variable.

Firm location might influence firm innovation as different locations provide various resources and opportunities for firms. It was measured by a dummy variable [75]. We measured it as 1 when a firm is in first-tier cities, including Beijing, Shanghai, Guangzhou and Shenzhen; otherwise, it was coded as 0.

Industry may influence a firm’s cognition and its needs for business model innovation [5]. It was measured by a dummy variable. We coded it as 1 when the firm belongs to industries including materials, consumer discretionary, healthcare and energy; otherwise, it was coded as 0.

Financial leverage reflects the degree of financial risk of a firm [76]. It was measured by a dummy variable. When the financial leverage of a firm is higher than the mean value of the financial leverage of all sample firms, the value was coded as 1; otherwise, it was coded as 0.

Operating leverage reflects the degree of operating risk of a firm [77]. The method to measure it is similar to that of financial leverage.

### 3.4 Statistical techniques

Following Hox (1994) [78], we tested the direct effects by hierarchical regression analysis, using SPSS 24 software. Following Hayes (2018) [79], we tested the chain-mediating effects by a bias-corrected bootstrapping procedure, using PROCESS v. 3.3.

## 4 Empirical results

### 4.1 Descriptive statistics and correlations

Table 2 shows the mean, standard deviation, and correlation coefficient of the variables. We found a positive and significant relationship between (a) R&D effort and collaboration breadth *(r = 0*.*458*, *p <0*.*01)*, (b) collaboration breadth and collaboration depth *(r = 0*.*579*, *p < 0*.*01)*, and (c) collaboration depth and business model innovation *(r = 0*.*552*, *p < 0*.*01)*. We also found that (a) R&D effort is positively related to collaboration depth *(r = 0*.*504*, *p < 0*.*01)*, (b) R&D effort is positively associated with business model innovation *(r = 0*.*554*, *p < 0*.*01)*, and (c) collaboration breadth is positively related to business model innovation *(r = 0*.*380*, *p < 0*.*01)*. Those findings provided preliminary evidence for the hypotheses proposed in our study.

**Table 2:** Descriptive statistics and correlations.

Variables	Mean	S.D.	1	2	3	4	5	6	7	8	9	10	11
1 Firm Ownership	0.610	0.491	1										
2 Firm Age	18.910	5.015	-0.066	1									
3 Firm Size (Ln)	10.149	1.079	0.052	-0.225**	1								
4 Firm Location	0.430	0.497	0.077	-0.330***	0.335***	1							
5 Industry	0.550	0.500	-0.111	-0.328***	-0.109	0.211**	1						
6 Financial Leverage	0.220	0.419	0.014	-0.047	0.009	0.003	-0.134	1					
7 Operating Leverage	0.170	0.378	0.017	-0.100	0.203*	0.125	0.008	0.233**	1				
8 R&D Effort	0.063	0.053	-0.215**	0.024	-0.471***	0.084	0.253**	-0.187*	0.012	1			
9 Collaboration Breadth	6.060	1.618	-0.022	0.124	-0.055	0.019	0.115	-0.228**	-0.123	0.458***	1		
10 Collaboration Depth	2.520	1.721	-0.111	0.140	-0.201*	-0.023	-0.014	-0.059	0.011	0.504***	0.579***	1	
11 Business Model Innovation	0.418	0.127	-0.347***	0.257**	-0.276***	-0.139	-0.125	-0.208**	0.028	0.554***	0.380***	0.552***	1

Notes:

*p < 0.1;

**p < 0.05;

***p < 0.01 (two-tailed tests).

Additionally, the highest correlation value among all variables is 0.579, far below the threshold value of 0.75 [80], suggesting no serious multicollinearity problem within the dataset. As shown in Table 3, it is confirmed again by the variance inflation factors (VIFs) of all variables, well below ten, the recommended threshold value [81].

**Table 3:** The results of hierarchical regression analysis.

Variables	Business Model Innovation		Collaboration Breadth		Collaboration Depth		Business Model Innovation	
	Model 1		Model 2		Model 3		Model 4	
	β	t-value	β	t-value	β	t-value	β	t-value
Constant	0.697***	3.869	-0.054	-0.258	0.151	0.797	0.662***	3.890
***Control Variables***								
Firm Ownership	-0.497***	-3.019	0.254	1.341	-0.099	-0.565	-0.512***	-3.269
Firm Age	0.118	1.347	0.169*	1.673	0.020	0.218	0.082	0.983
Firm Size (Ln)	0.003	0.031	0.343***	2.892	-0.053	-0.462	-0.042	-0.410
Firm Location	-0.171	-0.936	-0.197	-0.939	-0.018	-0.092	-0.131	-0.759
Industry	-0.507***	-2.895	0.158	0.784	-0.275	-1.485	-0.454***	-2.709
Financial Leverage	-0.362*	-1.823	-0.143	-0.626	0.176	0.841	-0.389**	-2.068
Operating Leverage	0.225	1.028	-0.424*	-1.680	0.171	0.729	0.249	1.187
***Main Variables***								
R&D Effort	0.542***	5.480	0.622***	5.450	0.299**	2.469	0.345***	3.081
Collaboration Breadth					0.476***	4.803	0.035	0.355
Collaboration Depth							0.295***	3.023
***Goodness-of-fit***								
R^2^	0.492		0.325		0.443		0.561	
Adj R^2^	0.444		0.261		0.384		0.508	
F	10.300***		5.111***		7.432***		10.621***	
Maximum VIF	1.776		1.776		2.211		2.372	
Durbin-Wastson	1.980		2.241		2.023		2.041	

Notes:

*p < 0.1;

**p < 0.05;

***p < 0.01 (two-tailed tests).

### 4.2 Hypotheses tests

**Fig 2:**
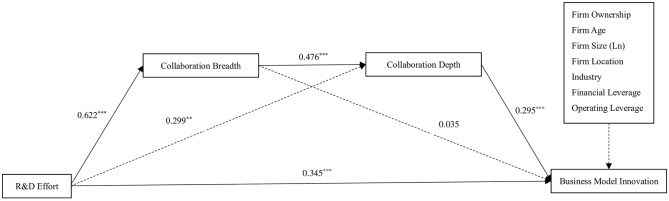
The fitted model of our study. **p < 0.05, ***p < 0.01 (two-tailed tests).

The hypotheses advanced in the study were tested through hierarchical regression analysis using SPSS 24 software. The results are shown in Table 3. The result of Model 1 shows that R&D effort positively influences business model innovation *(β = 0*.*542*, *p < 0*.*01)*, thus supporting Hypothesis 1. The result of Model 2 indicates that R&D effort positively affects collaboration breadth *(β = 0*.*622*, *p < 0*.*01)*, thereby supporting Hypothesis 2. According to Model 3, there is a significantly positive relationship between collaboration breadth and collaboration depth *(β = 0*.*476*, *p < 0*.*01)*, thus supporting Hypothesis 3. Moreover, Model 4 shows a positive effect of collaboration depth on business model innovation *(β = 0*.*295*, *p < 0*.*01)*, thereby supporting Hypothesis 4. The above results reveal the possibility of an indirect effect of R&D effort on business model innovation through the mediating role of collaboration breadth and depth in sequence. The path coefficients of the whole conceptual model are presented in Fig 2.

Table 4 shows the results of the chain mediation model tested by the bias-corrected bootstrapping procedure. There are three indirect effects between R&D effort and business model innovation. In detail, (a) the indirect effect via collaboration breadth is insignificant *(estimate = 0*.*022*, *95% CI = [-0*.*104*, *0*.*147])*, (b) the indirect effect via collaboration depth is significant *(estimate = 0*.*088*, *95% CI = [0*.*012*, *0*.*237])*, and (c) the chain-mediating effect of collaboration breadth and depth is significant *(estimate = 0*.*087*, *95% CI = [0*.*025*, *0*.*167])*. Hence, Hypothesis 5 is also supported.

**Table 4:** The results of the bootstrapping test.

Paths	Estimate	BootSE	95%CI	
			BootLLCI	BootULCI
*Direct Effect (dir)*: RDE → BMI	0.345	0.102	0.142	0.550
*Indirect Effect*				
(ind1) RDE → CB → BMI	0.022	0.062	-0.104	0.147
(ind2) RDE → CD → BMI	0.088	0.057	0.012	0.237
(ind3) RDE → CB → CD → BMI	0.087	0.036	0.025	0.167
*Total Indirect Effect*: ind1+ind2+ind3	0.197	0.081	0.064	0.384
*Total Effect*: dir+ind1+ind2+ind3	0.542	0.093	0.381	0.750

Notes: RDE = R&D Effort; CB = collaboration Breadth; CD = Collaboration Depth; BMI = Business Model Innovation.

### 4.3 Robustness tests

We analyzed the robustness of the above findings in two different ways. The first was to change the sample size, i.e., randomly selecting a subsample (N = 70). The second way was to replace a variable, i.e., using the percentage of highly skilled R&D workers to measure R&D effort. The results of robustness tests are presented in Table 5, showing that they are consistent with the initial findings. Therefore, we concluded that our findings are robust.

**Table 5:** Robustness tests.

Hypotheses	Paths	Changing sample size				Replacing variable			
		β		t-value		β		t-value	
H1	RDE→BMI	0.560***		4.599		0.534***		5.772	
H2	RDE→CB	0.775***		6.118		0.458***		3.967	
H3	CB→CD	0.418***		3.166		0.465***		5.247	
H4	CD→BMI	0.315***		2.752		0.242**		2.385	
		Estimate	BootSE	95%CI		Estimate	BootSE	95%CI	
				BootLLCI	BootULCI			BootLLCI	BootULCI
H5	RDE→CB→CD→BMI	0.102	0.050	0.013	0.212	0.051	0.026	0.008	0.110

Notes:

**p < 0.05;

***p < 0.01 (two-tailed tests).

## 5 Discussion

### 5.1 Theoretical implications

The theoretical contributions of the present study are threefold. First, this study examines the relationship between R&D effort and business model innovation. In fact, to our knowledge, we offered the first empirical evidence of how business model innovation is carried out under firms’ effort in R&D. The literature review that is performed, shows that neither the relationship nor the influencing mechanism of R&D effort on business model innovation has been previously discussed. We concluded that R&D effort is conductive to business model innovation, thereby extending the research on the antecedents of business model innovation. The antecedents of business model innovation have been explored in many aspects, such as organizational search [82], big data analytics capabilities [83] and strategic orientation [84]; however, few researchers have paid attention to the role of firms’ R&D effort. Similarly, our results also enrich the research on the outcomes of R&D effort. Most of the outcome variables in existing research on R&D effort have mainly focused on the performance of total innovation activities [19, 85, 86], while innovative behavior has rarely been involved.

Second, it contributes to the literature on external collaboration. According to past studies, collaboration breadth and depth are generally discussed as a pair of parallel constructs. For instance, setting them as two different strategies, Zhang, Yuan and Zhang (2022) explored their impacts on growth of new technology-based firms [87]. Similarly, Jang, Ko, Chung and Woo (2023) investigated their effects on product and process innovation [88]. In contrast, we argued that the two are neither orthogonal nor mutually exclusive. They can affect and complement each other. Considering that little prior work has explored their relationship, we demonstrated from the perspective of attention theory that the expansion of collaboration breadth is conducive to the improvement of collaboration depth. These findings open the black box of the relationship between collaboration breadth and depth, which enriches the open innovation field.

Finally, this study reveals the inside-out influencing mechanism of R&D effort on business model innovation from the perspective of absorptive capacity, addressing the necessity of taking into account the positive role of external collaboration. In most previous studies, both R&D effort and external collaboration have been positioned as direct predictors or moderators of firm innovation and other outcomes. Little attention has been given to the potential mediating mechanism. To cover the gap, we took both collaboration breadth and collaboration depth as mediators to explore the internal influencing mechanism of R&D effort on business model innovation. Following the R&D-PAC-RAC-Innovation link, the results of the present study demonstrate the chain-mediating effect of collaboration breadth and collaboration depth on the relationship between R&D effort and business model innovation. Our findings may help researchers to deepen their understanding of the internal mechanism through which R&D effort affects business model innovation; moreover, they provide a new perspective to investigate how business model innovation is triggered in the context of R&D dominant culture, and how to effectively combine the business strategy and technological strategy in a firm.

### 5.2 Practical implications

This study has important practical implications for managers. First, to achieve business model innovation, enterprises are suggested to put much effort into R&D. R&D effort can provide internal resource bases for business model innovation. Therefore, top managers should take some specific measures from the R&D perspective, such as increasing R&D expenditure, hiring enough R&D talent, building excellent R&D teams and developing a perfect R&D strategic system, to satisfy the requirements of business model innovation.

Second, enterprises are also recommended to promote many collaborative activities with external organizations. Through external collaboration, firms need to obtain advanced knowledge, gain updated information and learn new technologies and business thoughts, the combination of which provides new inspiration for enterprises to carry out business model innovation. To better promote collaborative communications, enterprises should cultivate an open and collaborative culture, develop online cooperative platforms, build cooperative alliances and so on.

Finally, firms are advised to follow the R&D-PAC-RAC-Innovation path to achieve the goal of business model innovation. That is, firms may follow three steps to make efforts. First, they should devote themselves to R&D activities to lay the foundation of external collaboration. Second, on the basis of technological advantages, they should build collaborative networks and develop their collaborators. Third, on the basis of broad collaborations, they need to construct strategic and deep collaborations for effective actions about resource integration, knowledge sharing, and win-win business. Our study reminds managers to gradually shift their attention from numerous collaborative relationships to several deep collaborations with VRIN characteristics. Through R&D-based collaborations, new business models can be effectively developed.

This study also has practical implications for governments. To some extent, our study could lead Chinese central and local governments to make better decisions about firms’ sustainable growth. On the one hand, governments are suggested to set some policies to stimulate firm innovation, such as allocating R&D subsidies, increasing benefits for introducing innovative talents and creating a great innovative business environment. On the other hand, governments are also encouraged to take measures to promote firm collaboration with external organizations, such as establishing cooperative funds, strengthening the construction of cooperation platforms and improving the tax incentives of collaborative projects.

### 5.3 Limitations and future research

Our research has some limitations. First, our database was collected at one point in time, but the process of business model innovation is really a longitudinal process [10]. The cross-sectional data used in this study can only reflect the correlations between the considered variables, but cannot infer their causal relationships. The collection of longitudinal data is recommended in the future. Second, the fact that our study focuses on firms in China limits the generalizability of our results. Future research should therefore conduct cross-country analyses to raise the external validity and robustness of our conclusions. Third, the self-developed measurement of business model innovation may be flawed and inadequate, so it still needs more tests and improvements. Future research can adopt questionnaire surveys or interviews with executives to capture facets of business model innovation. Fourth, although the sample size meets the requirement of regression analysis and mediating effect test for parameter estimation, it is still insufficient compared with China’s large population and numerous companies. Hence, future research needs to expand the sample size. Finally, although our study has controlled for some environmental factors, they do not cover all the possible contextual differences capable of affecting the relationships examined in our conceptual model. Thus, the opportunity for future research should be given to the development of more control variables such as competitive intensity and firm hierarchy [5].

Regardless of the limitations described above, our study brings out some possible future research avenues. For example, it may be interesting to further investigate the role of different types of collaboration depth (e.g., vertical, horizontal and competitor collaboration depth) as mediating variables in the relationship between R&D effort and business model innovation. Likewise, it is also stimulating to develop the study by introducing some possible moderating variables from environmental (e.g., market dynamism, technological turbulence and competitive intensity), organizational (e.g., organizational openness, internal resources and capabilities) and individual (e.g., cognition and personality of top management) perspectives. In addition, a good idea is to explore the interaction of internal and external enablers.

### 5.4 Conclusion

Based on the logic of R&D-PAC-RAC-Innovation, our study explores for the first time how R&D effort stimulates business model innovation via the chain-mediating mechanism of external collaboration breadth and depth from the perspective of absorptive capacity. Our results support the following proposed hypotheses: (1) R&D effort positively affects business model innovation; (2) R&D effort positively influences collaboration breadth; (3) collaboration breadth positively stimulates collaboration depth; (4) collaboration depth positively affects business model innovation; and (5) collaboration breadth and depth play a chain-mediating role in the relationship between R&D effort and business model innovation. The results of the present study provide important insights for business model innovation research. Although previous studies have investigated the antecedents of business model innovation, to date a discussion of the inside-out mechanism of R&D-driven business model innovation is lacking. Our study has developed a novel theory to explain the complex influencing mechanism between R&D effort and business model innovation.

## Supporting information

S1 File (DOCX)

S1 Data (XLSX)
