# Supplementary material for: The impact of R&D effort on business model innovation: Evaluating chain mediation through collaboration breadth and depth
Source: PLoS One. 2023 Jun 5;18(6):e0286715. doi: 10.1371/journal.pone.0286715 (PMC10241366; doi:10.1371/journal.pone.0286715)
Supplement: S1 File — (DOCX) [file pone.0286715.s001.docx]

**Appendix 1. The scale for measuring collaboration breadth.**

| Collaborators | Coding rules (In study period, the observed firm developed collaborative activities with ……) | | |
| --- | --- | --- | --- |
|  | 1= “no or low degree” | 2= “medium degree” | 3= “high degree” |
| Suppliers | Less than 10 suppliers | 10 to 100 suppliers | More than 100 suppliers |
| Customers | No or a few | Many domestic customers | Many [foreign](javascript:;) customers |
| Competitors | No or a few | Many domestic competitors | Many [foreign](javascript:;) competitors |
| Governments | No or a few | Municipal, provincial and national governments | Many [foreign](javascript:;) governments |
| Universities and educational institutions | No or a few | Domestic universities ranking in 211 project or domestic educational institutes | Domestic universities ranking in 985 project or [foreign](javascript:;) universities and educational institutions |
| Consultancy firms | No or a few | Many domestic consultancy firms | Many [foreign](javascript:;) consultancy firms |
| Venture capital firms | No or a few | Many domestic venture capital firms | Many [foreign](javascript:;) venture capital firms |
| Trade fairs and exhibitions | No or a few | Many domestic trade fairs and exhibitions | Many [foreign](javascript:;) trade fairs and exhibitions |
| Others | No or a few | Many domestic other organizations | Many [foreign](javascript:;) other organizations |

**Appendix 2. The scale for measuring collaboration depth.**

| Collaborators | Coding rules (In study period, the observed firm developed collaborative activities with collaborators on the level of ……) | | |
| --- | --- | --- | --- |
|  | 1= “no or low degree” | 2= “medium degree” | 3= “high degree” |
| Suppliers | No or little knowledge collaboration | Strategic cooperation and awards for excellent ones | Building cooperative organizations |
| Customers | No or little knowledge collaboration | Strategic cooperation and awards for excellent ones | Building cooperative organizations |
| Competitors | No or little knowledge collaboration | Strategic cooperation | Building cooperative organizations |
| Governments | No or little knowledge collaboration | Strategic cooperation and obtaining government funds | Building cooperative organizations |
| Universities and educational institutions | No or little knowledge collaboration | Strategic cooperation | Building cooperative organizations |
| Consultancy firms | No or little knowledge collaboration | Strategic cooperation | Building cooperative organizations |
| Venture capital firms | No or little knowledge collaboration | Strategic cooperation and getting investment | Building cooperative organizations |
| Trade fairs and exhibitions | No or little knowledge collaboration | Participation | Hosting |
| Others | No or little knowledge collaboration | Strategic cooperation and awards for excellent ones | Building cooperative organizations |

**Appendix 3. Items for measuring novelty-centered business model innovation.**

A five-point scale, ranging from 1 (strongly disagree) to 3 (neutral) to 5 (high degree), was used to measure novelty-centered business model innovation.

1. The observed enterprise has established a global R&D center and created a global intelligent R&D platform.

2. The observed enterprise has advanced intelligent manufacturing system.

3. The observed enterprise has diversified, advanced and intelligent sales system for offline and online sales.

4. The observed enterprise has advanced, convenient and intelligent service system for dealing with pre-sale, sale and after-sale issues.

**Appendix 4. The indicators for measuring efficiency-centered business model innovation.**

The entropy-based TOPSIS method was used to evaluate the variable on basis of the following indicators.

| Dimension | Indicator | Measure |
| --- | --- | --- |
| Value creation | Current ratio | Current assets/Current liabilities |
|  | Equity-to-debt ratio | [Total](javascript:;) [owners'](javascript:;) [equity](javascript:;)/Total liabilities |
|  | Debt coverage ratio | Net cash flow from operating activities/Total liabilities |
| Value proposition | Inventory turnover | Operating costs/Average inventory |
|  | Accounts receivable turnover | Operating income/ Average accounts receivable |
|  | Total assets turnover | Operating income/Average total assets |
| Value capture | Net profit growth rate | Current year’s net profit/ Last year’s net profit -1 |
|  | Operating income growth rate | Current year’s operating income/ Last year’s operating income -1 |
|  | Operating profit ratio | Operating profit/Operating income |
